# Evaluation and genome-wide association study of saline–alkali tolerance in high-latitude rice resource populations

**DOI:** 10.3389/fgene.2025.1617034

**Published:** 2025-07-29

**Authors:** Rongsheng Wang, Tan Lv, Jingpeng Li, Juntao Ma, Yongli Wang, Lingwei Deng, Wan Li, Jun Zhang, Kun Li, Wei Zhang, Fengchen Mu, Guomin Zhang

**Affiliations:** ^1^ Biotechnology Research Institute, Heilongjiang Academy of Agricultural Sciences, Harbin, Heilongjiang, China; ^2^ Heilongjiang Laboratory of Crop and Livestock Molecular Breeding, Harbin, Heilongjiang, China; ^3^ College of Agriculture, Heilongjiang Bayi Agricultural University, Daqing, Heilongjiang, China; ^4^ Northeast Institute of Geography and Agroecology, Chinese Academy of Sciences, Changchun, Jilin, China; ^5^ Institute of Farming and Cultivation, Heilongjiang Academy of Agricultural Sciences, Harbin, Heilongjiang, China

**Keywords:** oryza sativa japonica, saline-alkali tolerance, nature resource population, salt tolerance index, membership function, GWAS

## Abstract

**Introduction:**

China is the world’s third largest saline–alkali land country, and the breeding of salt-tolerant rice varieties has always been a key focus of rice breeders. Screening and identifying salt-tolerant varieties and exploring related genes are essential for breeding.

**Methods:**

In this study, 450 high-latitude resource populations were planted on natural saline–alkali soil for 2 years under 2 treatments. The comprehensive agronomic traits of the populations were evaluated. The principal component and cluster analyses were used to preliminarily group the phenotypes, and individual phenotypes were comprehensively scored and ranked to identify the top 40 saline–alkali tolerant varieties each year.

**Results:**

Notably, S321 and S19 were the most saline-alkali tolerant varieties each year. Genome-wide association studies identified one saline–alkali-related position near 6,636,119 bp on chromosome 8 and another near 23,311,931 bp on chromosome 11. Os08g0214233 and Os11g0604900 were the nearest genes from the identified positions, respectively. Gene annotation was used to further screen the polymorphic sites in the associated regions, identifying 17 and 48 genes with 593 variants, including 56 polymorphic sites located in exons.

**Discussion:**

This study provided candidate gene loci for the fine mapping of saline–alkali tolerance genes and offered excellent resistant rice resources for the molecular improvement of varieties.

## 1 Introduction

With global warming, the saline–alkali land continues to increase at a rate of 1 × 10^6^ to 1.5 × 10^6^ hm^2^ per year (Shahid et al., 2018). China is the world’s third largest saline–alkali soil country, with a total saline–alkali land area of about 100 million hm^2^, mainly distributed in the northeast, north, northwest, and other regions of China (Yang, 2006; Li et al., 2014). Studies have shown that using salt-tolerant rice varieties with appropriate farming practices can improve saline–alkali land (Wang et al., 2024). However, studies on rice’s regulatory mechanisms under saline–alkali stress and related genes were limited, hindering the full potential of salt-tolerant rice and making it difficult to establish a complete set of salt-tolerant rice cultivation techniques to achieve large-scale production and maximize benefits (Moeljopawiro and Ikehashi, 1981; Qin et al., 2020).

Recent studies in China and abroad have explored the molecular mechanisms of salt and alkali tolerance in plants such as rice. The regulatory mechanisms of rice include the following: 1. Organic osmotic penetration (Shen et al., 1997; Chen et al., 2011), such as the induction of the expression of multiple genes, including *OsP5CS1*, *OsTPS1*, *OsTPP*, and *TPS*, under salt stress, leading to the accumulation of organic substances such as proline, betaine, and trehalose, and increasing rice’s tolerance to stress (Sripinyowanich et al., 2013; Ge et al., 2008; Li et al., 2011). 2. The regulation of the redox equilibrium is achieved through antioxidative enzymes. When plants receive stress signals, these signals activate pathways that lead to the synthesis of reactive oxygen species (ROS). Excessive accumulation of ROS can lead to toxic reactions, such as lipid peroxidation in plants. To counteract this toxicity, plants need to synthesize their own antioxidative enzymes (Nadarajah, 2020; Xiao et al., 2021). Studies showed that a series of antioxidative enzyme genes, including *OsAPX2*, *OsAPXb*, and *OsAPx8*, were all involved in regulating saline–alkali stress in rice (Zhang et al., 2013; Sofo et al., 2015). 3. High salt and alkali concentrations can regulate the synthesis response of plant hormones such as auxin, gibberellin, brassinosteroids, and strigolactones, which regulate rice’s salt and alkali tolerance through a series of signal transductions (Wang et al., 2016; Zhao et al., 2021). In addition, plants can respond to salt and alkali stress by regulating the absorption and transport of ions in the plant (Wu, 2018; Wang et al., 2022). Genes related to the absorption and transport channels of Na^+^ and K^+^ ions in rice, such as *OsHKT1*, *OsHKT2*, *OsAKT1*, and *OsKCO1*, regulate rice’s tolerance to salt and alkali stress to a certain extent (Kumar et al., 2013; Campbell et al., 2017; Wei et al., 2021; Park et al., 2016).

With the rise of genomics research, many saline–alkali-tolerant genes have been discovered in recent studies using methods such as genome-wide association studies (GWAS) and bulked segregant analyses (Yano et al., 2016; Fekih et al., 2013; Takagi et al., 2013). Based on genome resequencing, these methods have high detection accuracy and rich variation within populations, making them particularly suitable for discovering genes related to quantitative traits (Abe et al., 2012; Kang et al., 2008). Northeast China has more than 3.2 × 10^6^ hm^2^ of saline–alkali land, concentrated in the Songnen Plain, which has one of the world’s three largest saline–alkali soils (Wang et al., 2009). Therefore, the discovery of saline–alkali tolerant genes in the japonica rice population in this region is of great significance for improving rice variety resistance and increasing grain yield.

Evaluating saline–alkali-tolerant rice varieties is crucial in comprehensively evaluating the complex yield-related traits and obtaining phenotypic data representing the saline–alkali resistance of the varieties. In plant abiotic stress research, the salt tolerance index is usually chosen as the phenotypic evaluation parameter (Masuda et al., 2021; Kumawat et al., 2018). However, it is easily influenced by environmental factors when dealing with multi-phenotype analysis. Therefore, applying the membership function method to calculate comprehensive scores can better evaluate the saline–alkali resistance of materials through algorithm improvement (Fan et al., 2023). In this study, 450 high-latitude rice resources were planted in 2 different saline–alkali levels for 2 consecutive years. Nine yield-related traits, including plant dry weight, heading date, panicle weight, panicle length, plant height, tiller number, grain number, seed setting rate and thousand grain weight, were investigated. The membership function method was used to comprehensively evaluate the saline–alkali resistance of the population, aiming to gain a deeper understanding of the resistance traits in these resource populations. Additionally, GWAS was conducted to screen for saline–alkali tolerance genes by combining comprehensive evaluation scores with population resequencing data. This approach further elucidated the saline–alkali resistance loci in the population, providing new insights for future gene discovery and the selection of gene donor varieties for resistance breeding.

## 2 Materials and methods

### 2.1 Study materials

The research panel for this study consisted of 450 samples of high-latitude japonica natural resources obtained from Jilin, Liaoning, and Heilongjiang provinces in China, as well as surrounding countries and regions. This mainly included cultivars and a small number of landraces. These introduced varieties matured and were harvested naturally in the study areas. Detailed information about the study population was previously introduced in the research of our project group, and the selection in this study was based on the field growth and phenotype data of the materials (Zhang et al., 2021).

### 2.2 Study design and phenotype investigation

The materials were cultivated at the Da’an Alkaline Land Ecological Experimental Station of the Chinese Academy of Sciences, Bajiazi Village, Honggangzi Township, Da’an City, Jilin Province, China (124.2926°E, 45.5070°N). Plot 1 had mild saline–alkali soil [pH 8.03, electrial conductivity (EC) 0.458 mS/cm, and exchange sodium percentage (ESP) 8.52%], serving as the control (CK). Plot 2 had moderate saline–alkali soil (pH 8.52, EC 0.59 mS/cm, and ESP 17.85%), serving as the stress field (AK). Both study locations were irrigated with river water and had been used for rice cultivation for several years, without planting other crops.

The materials were cultivated in the CK and AK fields in 2016 and 2017 for two consecutive years. First, seedling cultivation was carried out using the greenhouse dry seedling method, and the seedings were transplanted into the field after about 35 days. The field studies were conducted using a completely randomized block design, with each material being planted in a single row of 20 plants and spacing of 13 cm between plants and 30 cm between rows. The water management methods used in the two fields were consistent with those used in other local paddy fields.

All agronomic traits were measured according to the standards set forth in the Standard Evaluation System for Rice (International Rice Research Institute, 2002), including days to heading (DH), dry weight per plant (DW), plant height (PH), tiller number per plant (TN), grain number per plant (GN), thousand grain weigh (TGW) and unfilled grain number (UG). Phenotypes were randomly selected from five plants in each accession, and the average was calculated. The seed setting rate was calculated as SR = GN/(GN + UG). The panicle length (PL) and panicle weight (PW) were measured from 20 randomly selected panicles per variety, and the average was recorded as the phenotype.

### 2.3 Phenotypic statistical analysis

All survey data were entered into MS Excel for classification and summarization, with the calculation of average values. The basic data statistical analysis was conducted using the base package in R (Milano et al., 2019), whereas data visualization was performed using R packages such as ggplot2, ggpubr, and ggsignif (Wickham, 2016; Constantin and Patil, 2021; Kassambara, 2023).

#### 2.3.1 Genetic diversity analysis

In this study, the genetic diversity analysis of phenotype data was conducted using the Shannon–Weaver diversity index (H′). The index was calculated using the following formula:

$$H^{\prime }=-\sum \left(\text{Pi}\right)\left(\ln\text{Pi}\right)$$

where $P_{i}=N_{i}/N$ . Ni represents the number of individuals in each group and N is the total number of individuals in each phenotype; the calculation used the natural constant (e) as the base and was performed after excluding missing individuals (Keylock, 2005). The analysis was carried out using the vegan package in R (Oksanen et al., 2022).

#### 2.3.2 Correlation, cluster, and principal component analyses

Different years and environmental conditions were analyzed separately for correlation analysis. The Pearson correlation coefficient matrix was calculated using the corr.test function in the psych/R package (William, 2024). A correlation heat map was generated using the ggcorrplot package in R. Before conducting cluster and principal component analyses, the phenotype data were standardized to avoid the influence of varying measurement scales on subsequent analyses. For cluster analysis, the Euclidean distance between samples was calculated based on the standardized phenotype data. Hierarchical clustering was then performed using the Ward minimum variance method with functions from both the stat and psych packages in R. The principal component analysis involved calculating the standard deviation and variance explained by each principal component. The number of principal components and clusters was determined based on the eigenvalues of each principal component and cumulative variance percentage. Finally, the standardized loadings matrix of phenotypic traits corresponding to each principal component was derived from the correlation matrix.

#### 2.3.3 Calculation of broad-sense heritability

The lme4/R package was used to calculate the broad-sense heritability of phenotypic data across 2 years at two treatments. The phenotypic data were defined as factors, including varieties, treatments, years, and interactions between varieties and treatments, as well as varieties and years as random factors (Bates et al., 2015). The formula used to calculate heritability was as follows:

$$R=V_{A}/\left(V_{A}+\frac{V_{\text{AL}}}{L}+\frac{V_{\text{AY}}}{Y}+\frac{V_{e}}{L\times Y}\right)$$

where VA represents the variance between varieties, VAL the interaction variance between varieties and treats, VAY the interaction variance between varieties and years, Ve the residual, L the number of experimental treatments, and Y the number of experimental years.

#### 2.3.4 Calculation of salt tolerance index (S) and membership function value (U)

First, the individual salt tolerance index was calculated based on the phenotype values across 2 years and two treatments using the formula $S_{i}=V_{\text{AK}}/V_{\text{CK}}\times 100\%$ . VAK represents the phenotype values of various traits of the individual in the AK environment, and VCK the phenotype values of various traits in the CK environment. The salt tolerance index matrix of the population was obtained for 2 years. The principal component analysis was performed on the salt tolerance index of each phenotype, and the score matrix of each principal component and the variance contribution rate PVi were obtained. The columns of the score matrix of each principal component were summarized, and the maximum value Smax and the minimum value Smin were calculated. Then, we used fuzzy mathematics to calculate the membership function (Ui) matrix as follows:

$$U_{i}=\frac{S_{i}-S_{\min}}{S_{\max}-S_{\min}}$$

Where, Si represents the score value of the ith principal component of the individual, Smax the maximum value in the score values of the ith principal component, and Smin the minimum value in the score values of the ith principal component. The weight of each principal component was calculated as: $W_{i}={\text{PV}}_{i}/\sum {\text{PV}}_{i}$ . To comprehensively evaluate the saline–alkali tolerance of the individual, a comprehensive calculation was performed on the membership function to obtain the comprehensive evaluation value of salt tolerance as: $D=\sum \left(U_{i}\times W\right)$ , where Ui is the membership function value of the ith principal component and PVi the variance contribution rate of the ith principal component.

### 2.4 GWAS and candidate gene discovery

The GWAS were conducted using the Genome Association and Prediction Integrated Tool (GAPIT) package in R, with the association model being the compressed mixed linear model (Wang and Zhang, 2021). A total of 189,019 SNPs were used in the GWAS. The confidence interval was calculated using Bonferroni correction as follows: –Log10 (P) ≥−Log10 (1/189,019) ≈ 5.28. The genotype data were obtained from the whole-genome resequencing data of our research group, with sequencing details referenced in a previous study (Zhang et al., 2021). Manhattan and Quantile-Quantile plots (QQ plots) were generated using the package CMplot in R (Version 4.5.1, https://github.com/YinLiLin/CMplot) (Li, 2024). The associated loci were annotated using the GenBank annotation database (2024-4-4, National Institute of Agrobiological Sciences) as a reference. SnpEff (Version 4.3T) software was used for annotating single-nucleotide polymorphisms (SNPs) at the associated loci (Cingolani et al., 2012).

## 3 Results

### 3.1 Analysis of differences in agronomic traits under different treatments

In 2016, the CK group showed coefficients of variation (CV) for nine phenotypic traits ranging from 7.65% to 35.86%. The smallest CV was for the heading date, indicating little variation in the heading date among varieties that could mature normally in high-latitude areas. The highest CV was for grain number (35.86%), indicating significant differences in this trait, which were linked to varying panicle type requirements in rice breeding across Heilongjiang Province. The genetic diversity for these phenotypes were not large, but it exceeded 6%, indicating substantial genetic variation. Traits such as 1000-grain weight, PL, and heading date showed the highest CV at 6.08. The CV in the AK group ranged from 6.48% to 31.79%. Similar to the CK group, the heading date and grain number in the AK group were the minimum and maximum values of the CV, respectively. The maximum genetic diversity was 6.08 for the DH phenotype (Supplementary Table S1).

**FIGURE 1:**
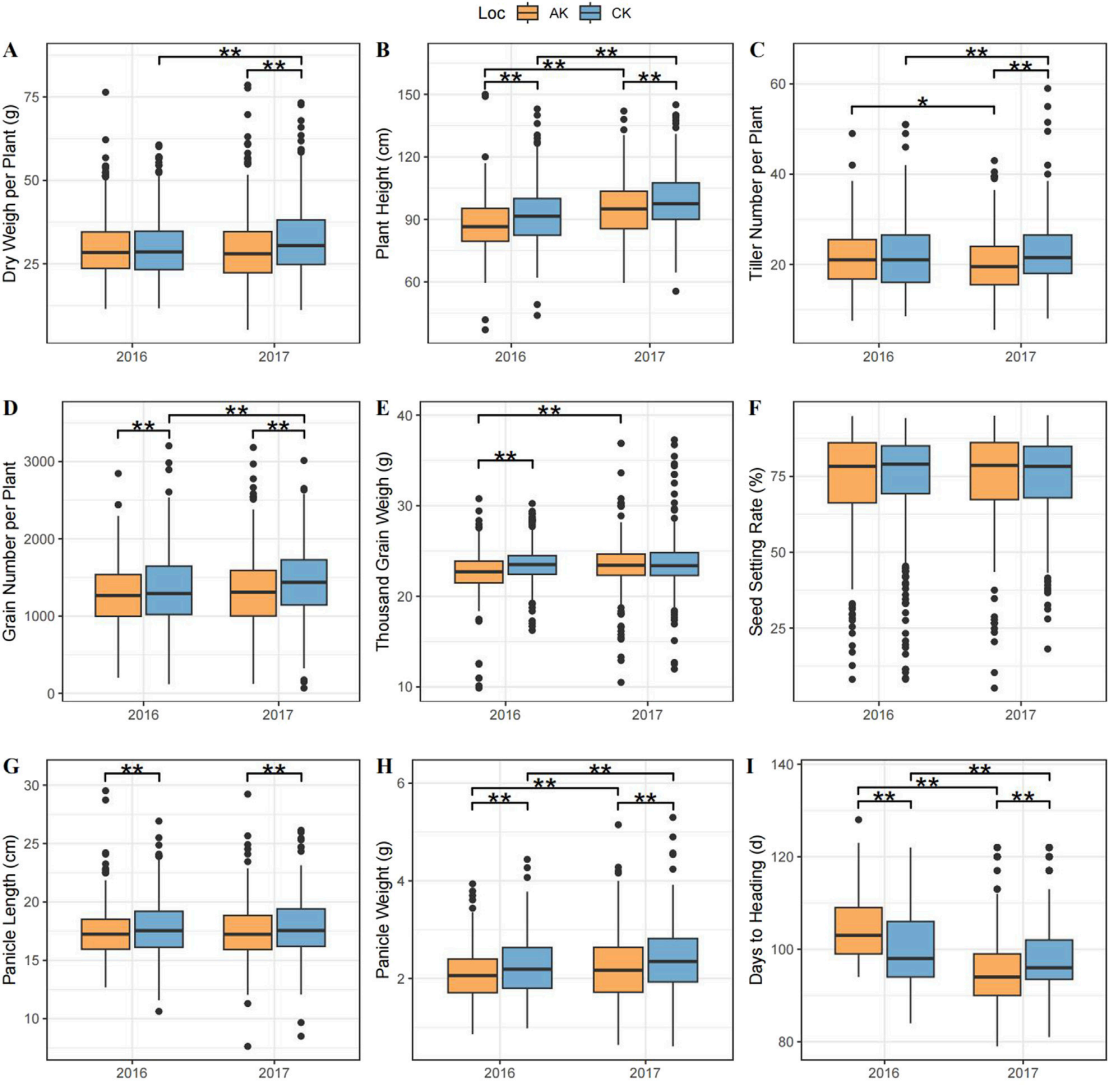
Box plot of interannual and phenotypic trait distributions under different treatments. **(A)** Dry weight per plant, **(B)** plant height, **(C)** tiller number per plant, **(D)** grain number per plant, **(E)** 1000-grain weight, **(F)** seed setting rate, **(G)** panicle length, **(H)** panicle weight, and **(I)** days to heading. The horizontal axis represents data from 2016 to 2017, and the vertical axis represents the distribution of values for each phenotype. The yellow box plot represents the AK group, and the blue box plot the CK group. ^**^ indicates a significant difference between the two groups at the 0.01 level, and ^*^ indicates a significant difference at the 0.05 level.

The investigation of phenotype showed that the change trend during 2 years was generally consistent. In the CK group, the CV ranged from 7.82% to 32.89%. The highest genetic diversity was observed in TGW and DH. In the AK group, the CV ranged from 8.10% to 36.89%, with the highest genetic diversity values observed in PH, TGW, PL, and DH, all at 6.08. In 2017, the maximum and minimum CV in both groups were DH and DW, respectively. However, unlike in 2016, the CV in DW was higher than that in GN (Figure 1; Supplementary Table S1).

In the same year, significant differences were observed in PH, GN, PL, and PW, with lower values in the AK group compared with the CK group. This suggested that plant growth in the AK group was inferior to that in the CK group, consistent with the expected experimental outcomes. Additionally, PH, TN, and PW showed significant differences across years under the same treatment, indicating that the traits might be influenced by annual variations in field conditions. However, no significant difference was observed in PL between years. DW and TGW showed significantly lower values in the AK group compared with the CK group in 1 year only, with significant differences observed between years under the same treatment. These variations could be attributed to environmental factors or interactions between yield traits. SR did not show significant differences between years or treatments, indicating the minimal impact of experimental treatments on seed setting rate. However, DH exhibited significant differences between years and treatments, showing opposite phenotype changes in different years. This indicated that different treatments across years were affected by different environmental factors, leading to an inconsistent variety of responses during heading.

### 3.2 Correlation analysis of various traits under different treatments

**FIGURE 2:**
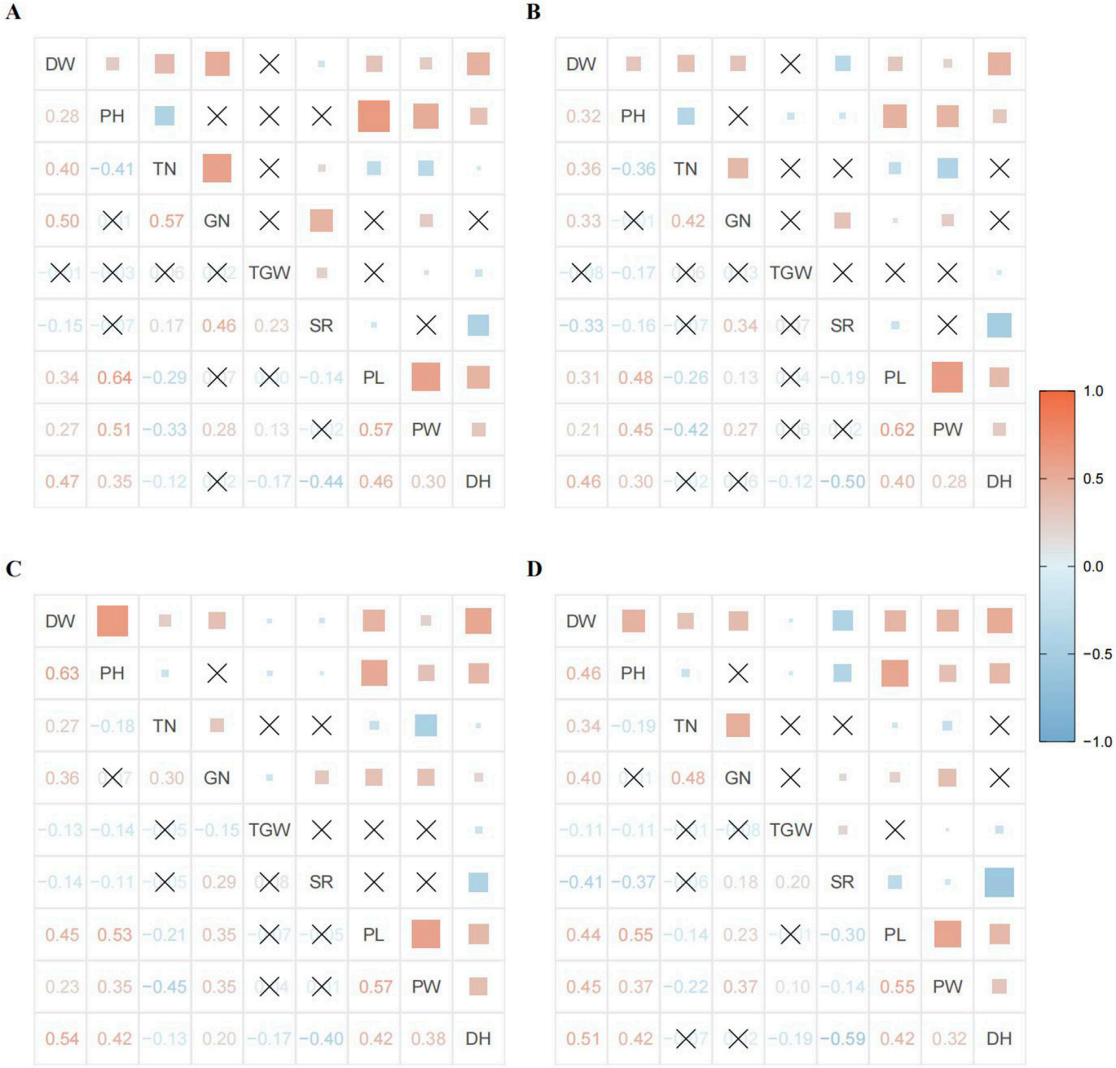
Interannual and inter-treatment correlation coefficient matrices and heat maps for different phenotypes: **(A)** 2016 CK; **(B)** 2016 AK; **(C)** 2017 CK; and **(D)** 2017 AK. The lower left of each image shows the correlation coefficient matrix, and the upper right shows the correlation heat map. The blue color indicates a negative correlation, and red indicates a positive correlation, the lighter the color, the weaker the correlation. The larger the colored square, the greater the absolute value of the correlation coefficient. A cross indicates no correlation or a tiny correlation.

We first conducted a correlation analysis to clarify the correlation among phenotype data under different environmental conditions and provide a basis for subsequent cluster and principal component analyses. The correlation between various traits showed a consistent overall trend, but individual traits were observed indicating differences in size under different treatments. Among these, DW was positively correlated with PH, TN, GN, PL, PW, and DH, but negatively correlated with SR. PH was mainly positively correlated with PL, PW, and DH, and negatively correlated with TN. TN exhibited a strong positive correlation with GN, negative correlations with PL and PW, and minimal correlations with TGW and DH. The correlation between GN and TGW, PL, and DH varied slightly between years and treatments, such as having a positive correlation with PL in 2017 but a lower correlation in 2016. GN demonstrated a strong positive correlation with SR and PW. SR showed a strong negative correlation with DH, indicating substantial differences in light response among the population materials, directly affecting the material’s seed setting rate. In addition, a strong positive correlation was observed between PL, PW, and DH (Figure 2). The correlation analysis also revealed that the selected phenotype traits in this study were not completely independent, and certain linear relationships were observed between them. This inherent linear relationship must also be considered in the subsequent analyses.

### 3.3 Cluster and principal component analyses under different treatments

We first used the Ward minimum variance method for hierarchical clustering to further clarify the inherent relationship of the phenotype of the experimental populations. The clustering tree analysis revealed slight differences in the classification of populations in different years and environments. For example, in the 2016 CK group, the clustering tree could be clearly divided into 3 clusters, each containing 110, 117, and 212 individuals, but no disagreement was reported in the AK group. The phenotype clustering of the 2017 population also confronted the same issue; on the one hand, it indicated a certain bias in the phenotype variation between years, possibly due to the influence of various factors such as light, temperature, and so on. On the other hand, the saline–alkali treatment of the experimental population might exacerbate the occurrence of variation, leading to greater uncertainty (Supplementary Figure S1).

Cluster analysis involves grouping a set of objects under study; however, it does not test statistical hypotheses. We calculated eigenvalues and eigenvectors of phenotype data in various environments and plotted scree plots to further clarify multiple phenotype data groupings and reduce data dimensions (Supplementary Figure S2). The eigenvalues and scree plots showed that the eigenvalues and standard deviations of the first three principal components were all greater than 1 under different treatments, except for the 2016 AK, where the first four components had eigenvalues and standard deviations greater than 1. For consistency in subsequent analysis, we used three principal components, with a cumulative proportion of variance ranging from 67.49% to 71.49%, representing all the data (Supplementary Table S2).

**FIGURE 3:**
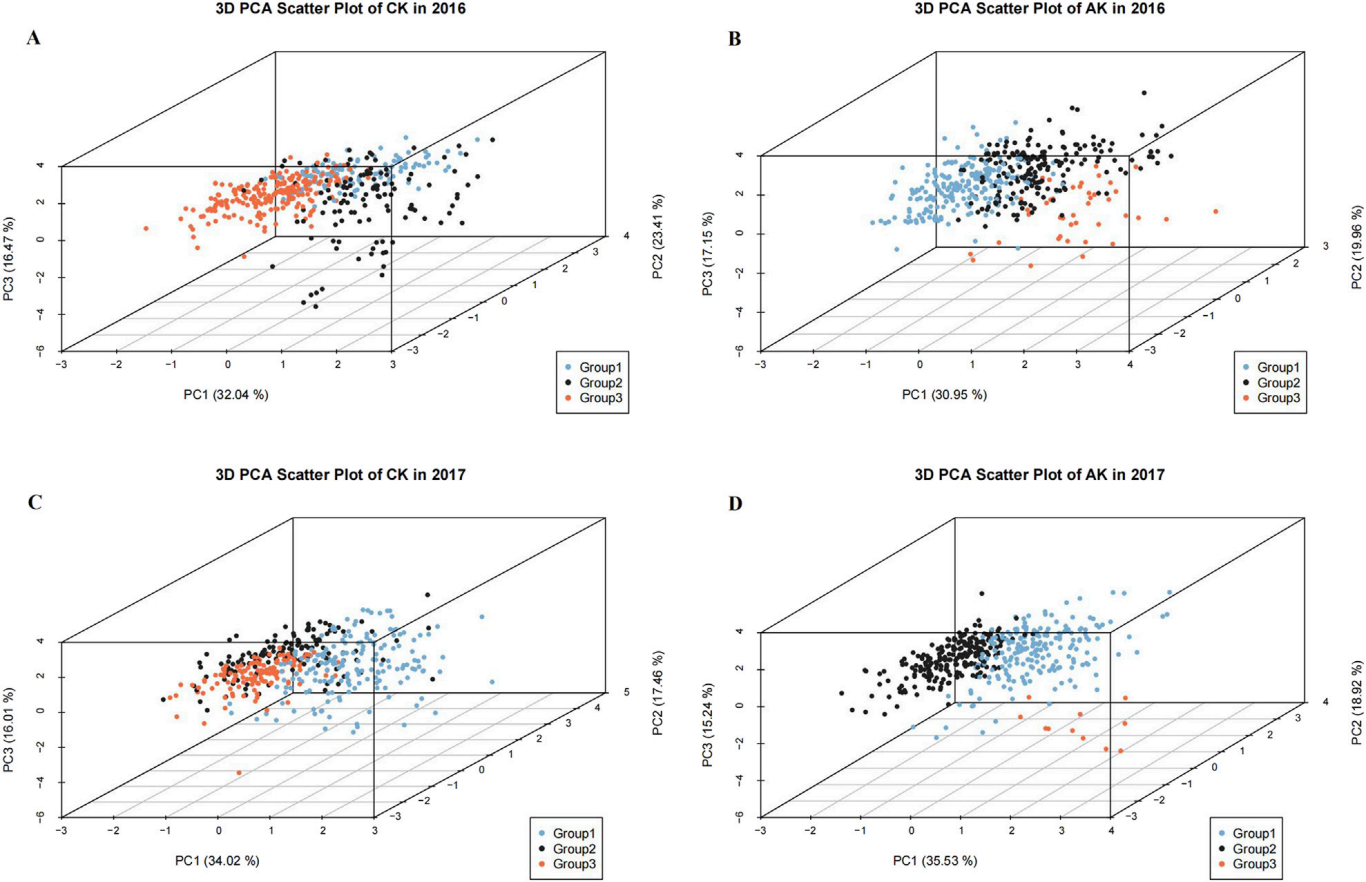
Scatter plots of the scores of the first three principal components under different treatments and different grouping: **(A)** 2016 CK; **(B)** 2016 AK; **(C)** 2017 CK; and **(D)** 2017 AK. The *x*-axis represents Principal Component 1, the *y*-axis represents Principal Component 2, and the *z*-axis represents Principal Component 3. Different colors represent different groups in the cluster analysis, with blue representing Group 1, black Group 2, and red Group 3.

The ingredient matrix showed that PH, PL, PW, and DH had larger coefficients in PC1, making them the principal influencing factors in this group. TN and SR had a larger coefficient in PC2 and PC3, respectively, indicating their importance in these groups. DW, GN, and TGW were divided into three different groups across treatments, indicating differences in the division of different principal components (Supplementary Table S3). Drawing a three-dimensional scatter plot of the principal component scores of each individual in the experiment can also visually display the division of the three principal components and the classification of the corresponding groups (Figure 3).

### 3.4 Analysis of genotype–environment interaction and selection of saline–alkali resistant varieties

We conducted a three-factor analysis of variance on all phenotype survey data to verify the phenotype variation and genotype–environment interaction in different treatment groups. The results showed a significant impact of population genotype on all traits, indicating genetic factors in response to different saline–alkali environments. TN, SR, and PL showed no effect of interannual factors, indicating that these traits were mainly determined by genetic factors. Soil salinity levels significantly impacted all traits except SR, indicating that the degree of salinity and alkalinity significantly impacted population phenotypes. Significant genotype–year interactions were observed in DW, PH, TGW, SR, PL, PW, and DH, whereas only PW showed a genotype–treatment interaction. Only an interaction between genotype and different treatments was observed in the PW phenotype. We estimated the heritability of population phenotypes to further analyze the proportion of genetic variation in total variation. The results showed that the heritability from high to low was in the following order: DH > PH > SR > PW > PL > TN > TGW > DW > GN (Table 1).

**TABLE 1:** Summary of three-factor analysis of variance results and heritability.

Traits	F (G)	F (Y)	F (L)	F (G × Y)	F (G × L)	R
DW(g)	2.80^a^	8.40^b^	18.54^a^	1.37^a^	1.08	0.4826
PH(cm)	8.49^a^	512.71^a^	133.76^a^	2.61^a^	1.15	0.6744
TN	2.81^a^	0.29	21.99^a^	1.13	0.98	0.5972
GN	1.85^a^	12.94^a^	31.58^a^	0.98	0.79	0.4678
TGW(g)	2.58^a^	24.01^a^	37.67^a^	1.26^b^	0.90	0.5102
SR (%)	4.28^a^	2.39	0.00	1.52^a^	0.97	0.6459
PL (cm)	5.43^a^	0.04	39.31^a^	2.09^a^	1.08	0.6007
PW(g)	5.00^a^	52.99^a^	81.51^a^	1.66^a^	1.28^b^	0.6131
DH(d)	9.96^a^	724.31^a^	25.73^a^	2.05^a^	0.24	0.7947

Note: F (G), genotype variance; F (G × Y), genotype × year interaction variance; F (G × L), genotype × treatment interaction variance; F (L), treatment variance; F (Y), year variance; R, broad-sense heritability.

^a^ Significant at the 0.001 level.

^b^ Significant at the 0.01 level.

We calculated the saline–alkali tolerance index for eight phenotype traits that showed significant differences in genetic and environmental factors so as to analyze the comprehensive effects of various traits on the saline–alkali resistance of varieties, and conducted the principal component analysis. The cumulative contribution rate of the first five principal components exceeded 80%. We calculated the membership function for all individuals in the first five principal components and used it with the variance contribution rate to determine comprehensive scores. In 2016, S321, S352, S41, S403, and S295 were the top five saline–alkali resistant varieties; in 2017, S19, S243, S197, S84, and S422 were the top five saline–alkali resistant varieties (Table 2). These can be used as candidate varieties for future field screening of saline–alkali resistant rice varieties or as basic experimental materials for exploring related resistance genes.

**TABLE 2:** List of the top 40 rice varieties in terms of salt and alkali tolerance comprehensive score ranking.

Rank	Accessions-2016	Scores	Accessions-2017	Scores	Rank	Accessions-2016	Scores	Accessions-2017	Scores
1	S321	0.7363	S19	0.7450	21	S25	0.5975	S215	0.5763
2	S352	0.6920	S243	0.6711	22	S15	0.5974	S339	0.5756
3	S41	0.6904	S197	0.6656	23	S412	0.5973	S250	0.5751
4	S403	0.6835	S84	0.6498	24	S404	0.5939	S196	0.5740
5	S295	0.6803	S422	0.6374	25	S308	0.5921	S124	0.5727
6	S253	0.6767	S18	0.6338	26	S82	0.5864	S311	0.5707
7	S21	0.6756	S170	0.6150	27	S260	0.5856	S337	0.5706
8	S410	0.6577	S32	0.6124	28	S270	0.5844	S245	0.5699
9	S415	0.6520	S156	0.6118	29	S46	0.5830	S45	0.5694
10	S397	0.6482	S56	0.6023	30	S61	0.5814	S144	0.5694
11	S256	0.6378	S209	0.6022	31	S383	0.5813	S303	0.5688
12	S393	0.6314	S246	0.5934	32	S340	0.5808	S86	0.5684
13	S78	0.6314	S21	0.5896	33	S196	0.5738	S20	0.5684
14	S461	0.6309	S294	0.5882	34	S79	0.5723	S232	0.5682
15	S391	0.6241	S139	0.5879	35	S362	0.5709	S16	0.5681
16	S317	0.6217	S211	0.5876	36	S34	0.5704	S223	0.5659
17	S259	0.6163	S25	0.5873	37	S385	0.5701	S67	0.5644
18	S380	0.6007	S203	0.5847	38	S359	0.5687	S26	0.5643
19	S378	0.5987	S340	0.5832	39	S320	0.5671	S37	0.5639
20	S289	0.5985	S216	0.5828	40	S268	0.5660	S248	0.5623

### 3.5 GWAS and candidate gene discovery

**FIGURE 4:**
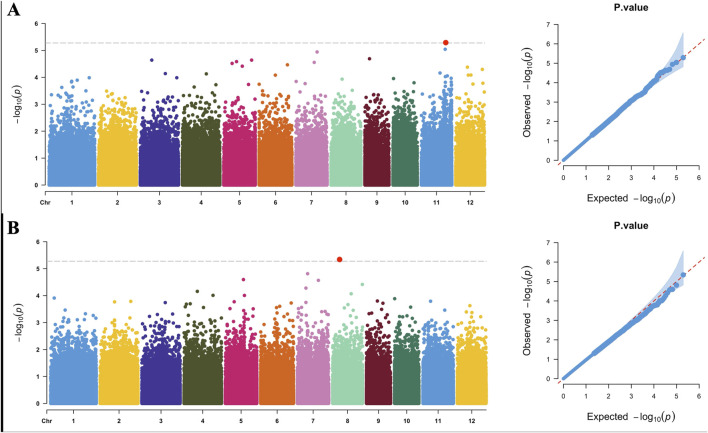
Manhattan and QQ plots of 2-year saline–alkali tolerance genome-wide association studies: **(A)** 2016 Manhattan plot (left) and QQ plot (right); **(B)** 2017 Manhattan plot (left) and QQ plot (right).

We conducted a GWAS using the saline–alkali tolerance comprehensive score as the phenotype (Supplementary Figure S3). The 2016 analysis identified a locus on chromosome 11 at 23,311,931 bp, and the 2017 analysis identified a locus on chromosome 8 at 6,636,119 bp (Figure 4). Reports on linkage disequilibrium in the temperate japonica rice population revealed that linkage disequilibrium decay rates were estimated at about 150 kb (Huang et al., 2010). We conducted gene screening and annotation analysis within a 300-kb range around these 2 associated loci (150 kb for each side), identifying 16 annotated genes on chromosome 8 with 282 polymorphic sites and 48 genes on chromosome 11 with 311 polymorphic sites (Table 3). A total of 593 variations were annotated, including 489 SNPs and 104 InDels, mainly located in intergenic regions, with 56 sites in exon regions (Supplementary Table S4). Annotation analysis of genes near the associated loci can identify clearer targets for subsequent gene mapping and expression screening, as well as candidate target sites for saline–alkali resistant breeding.

**TABLE 3:** Saline–alkali tolerance-associated markers and related variation statistics.

Marker	Chr	Position	P value	-LOG10 (P)	Nearest gene	Number of genes	Number of SNPs
S8_6636119	8	6,636,119	4.56e-06	−5.34	Os08g0214233 (8790 bp)	16	282
S11_23311931	11	23,311,931	5.14e-06	−5.29	Os11g0604900 (Intron)	48	311

## 4 Discussion

### 4.1 Selection of saline–alkali stress conditions

Different types of saline–alkali soils are formed under specific natural conditions, with saline soil containing chloride or sulfate salts and alkali soil containing carbonate or phosphate salts. Saline–alkali-tolerant plant breeding often uses salt concentration or pH as the stress condition, which may not reflect actual field environments. The most authentic phenotype feedback can be obtained by exploring gene functions under natural saline–alkali stress conditions, thus aiding in identifying practical key genes.

### 4.2 Phenotypic selection and gene identification in rice

Rice plants subjected to saline–alkali stress result in weaker plant growth, with different degrees of response in various phenotypes. However, the correlation and principal component analyses revealed no significant correlation between a single trait and saline–alkali stress. Hence, it is impossible to simply select a single trait to measure the saline–alkali resistance of rice varieties throughout the growth period. Previous studies combined the fuzzy mathematics theory to calculate the saline–alkali tolerance scores of various varieties based on saline–alkali tolerance index, revealing the strength of saline–alkali tolerance relationships (Fan et al., 2023). However, this method was influenced by environmental factors, selection of representative phenotypes, and so forth, leading to potential biases in the results. Therefore, further field experiments on these varieties are needed to promote the development and application of resistant varieties.

Many genes and QTLs associated with saline–alkali tolerance have been identified, with some located on chromosomes 8 and 11. OsNAC5 is the abiotic stress-responsive transcription factor located on chromosome 11, but it lies far from the associated region (Takasaki et al., 2010). OsFBDUF54 is another genes cloned for saline-alkali tolerance, situated more than 1.2 Mb away from the identified region (Li et al., 2020). In addition, numerous QTLs have been detected for various traits under saline–alkali stress conditions. Howerver, none of these contain the associated regions, including QTLs such as qDRW11, qSH11, and qRRN11 on chromosome 11 (Wang et al., 2012; Qi et al., 2009), and qDLRs8, qDSRs8, and qRL8 on chromosome 8 (Liang et al., 2015; Sabouri et al., 2009). qDM8 was a salt tolerance QTL identified in an F2:3 population based on shoot dry mass of. The associated markers on chromosome 8 in our study were located in this QTL. Further analysis is needed to verify their consistency.

### 4.3 Phenotypic evaluation of GWAS

GWAS typically uses analysis models for single phenotype association analysis, but the evaluation of abiotic stress often involves multiple phenotypes. Therefore, how to use statistical methods to evaluate multiple phenotype traits as a whole is a major issue that needs to be considered in the GWAS of abiotic stress. The comprehensive saline–alkali tolerance score is based on principal component analysis, calculated by summarizing several principal components with high contribution rates. It has been applied in studies of abiotic stresses such as heat tolerance, cold tolerance, and frost tolerance in various crops, ensuring the reliability of its use in association analysis. This study combined this phenotype with population genotype variation to conduct GWAS, aiming to obtain more representative saline–alkali tolerance functional gene loci for further gene discovery and breeding applications.

## 5 Conclusion

This study investigated and analyzed 9 phenotypes of 450 rice resource populations in low- and medium-saline–alkali fields. The population phenotype changes were basically consistent, with slight differences in genetic diversity. The resource populations were divided into three clusters based on hierarchical cluster analysis, but certain deviations were observed between different years. The principal component analysis showed that PH, PL, PW, and DH were the main influencing factors of phenotype under various treatments, providing a reference for subsequent gene–environment interactions. Considering the interaction between genotypes and environments in different years, we conducted variance analysis of each phenotype factor and calculated the heritability of each phenotype, with the heritability ranking from high to low as DH > PH > SR > PW > PL > TN > TGW > DW > GN.

Finally, we used the membership function method to calculate the comprehensive saline–alkali tolerance score of varieties based on the saline–alkali tolerance index. We obtained some candidate resources with good saline–alkali resistance in the population. Then, we used the population genotype data obtained earlier for GWAS and located a saline–alkali-associated region on chromosomes 8 and 11. The annotation analysis of the region revealed genomic variation, providing clear associated loci and candidate genes for further fine mapping of genes.

This study may provide more accurate basic data for exploring saline–alkali-resistant gene, and candidate gene resources for precise molecular improvement and breeding of saline–alkali-resistant rice varieties, thus promoting the development and utilization of saline–alkali land and increasing rice production.

## Data Availability

Raw reads of 450 accessions used in this study were a part of BioProject PRJCA000322 in the National Genomics Data Center (https://ngdc.cncb.ac.cn).
