# Supplementary material for: Evaluation and genome-wide association study of saline–alkali tolerance in high-latitude rice resource populations
Source: Front Genet. 2025 Jul 29;16:1617034. doi: 10.3389/fgene.2025.1617034 (PMC12339352; doi:10.3389/fgene.2025.1617034)
Supplement: Supplementary file 2 [file DataSheet1.docx]

Supplementary Figures

#
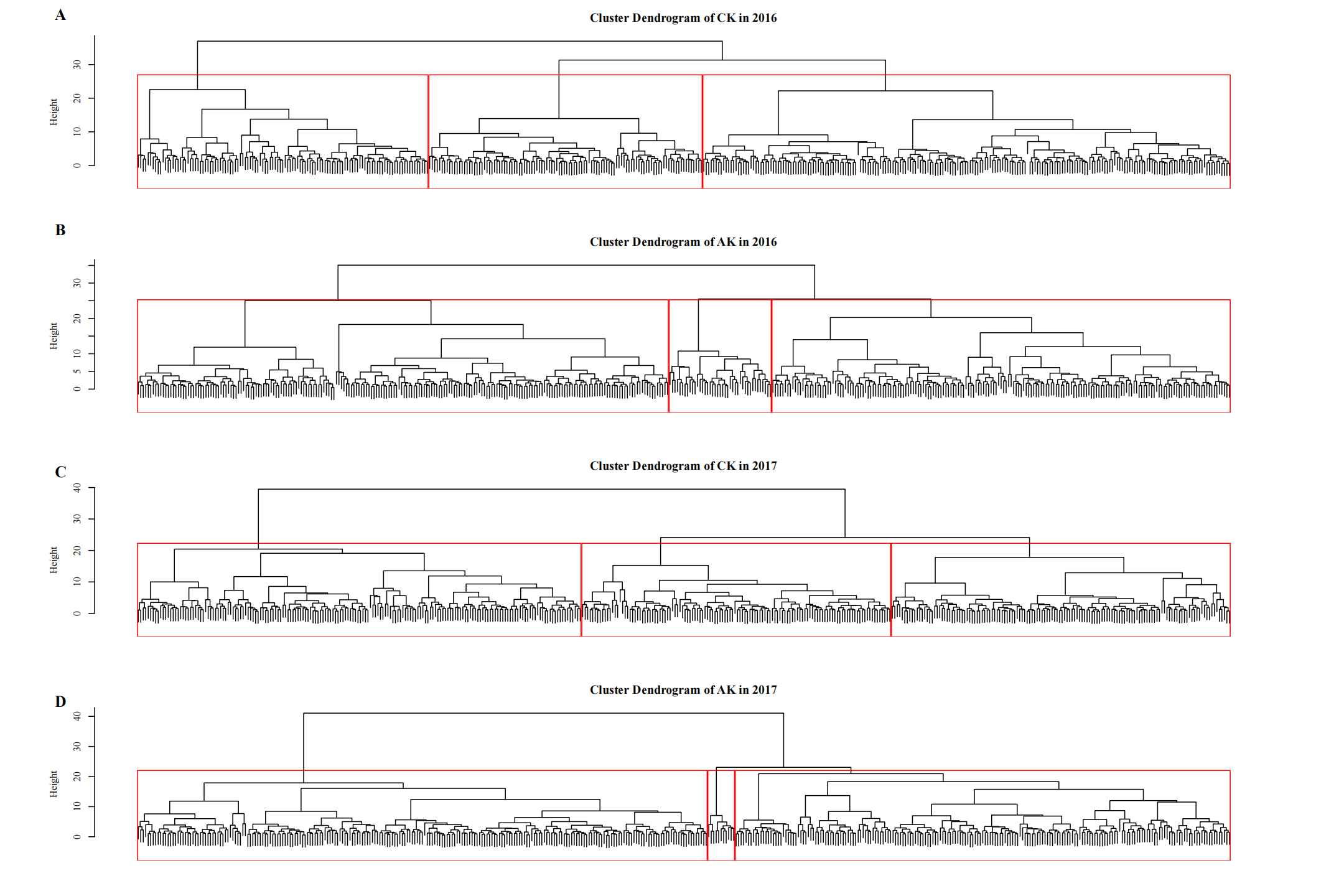
Supplementary Figures

**Figure S1.** Hierarchical clustering diagrams of different treatment groups: (A) 2016 CK; (B) 2016 AK; (C) 2017 CK; and (D) 2017 AK. The horizontal axis represents the experimental individuals, and the vertical axis represents the clustering tree height. The red border indicates the classification of the population into three categories.

**Figure S2.** Scree plot of principal component eigenvalues under various treatments: (A) 2016 CK; (B) 2016 AK; (C) 2017 CK; (D) and 2017 AK. The horizontal axis represents Components 1 to 9, and the vertical axis represents the eigenvalues of each principal component.


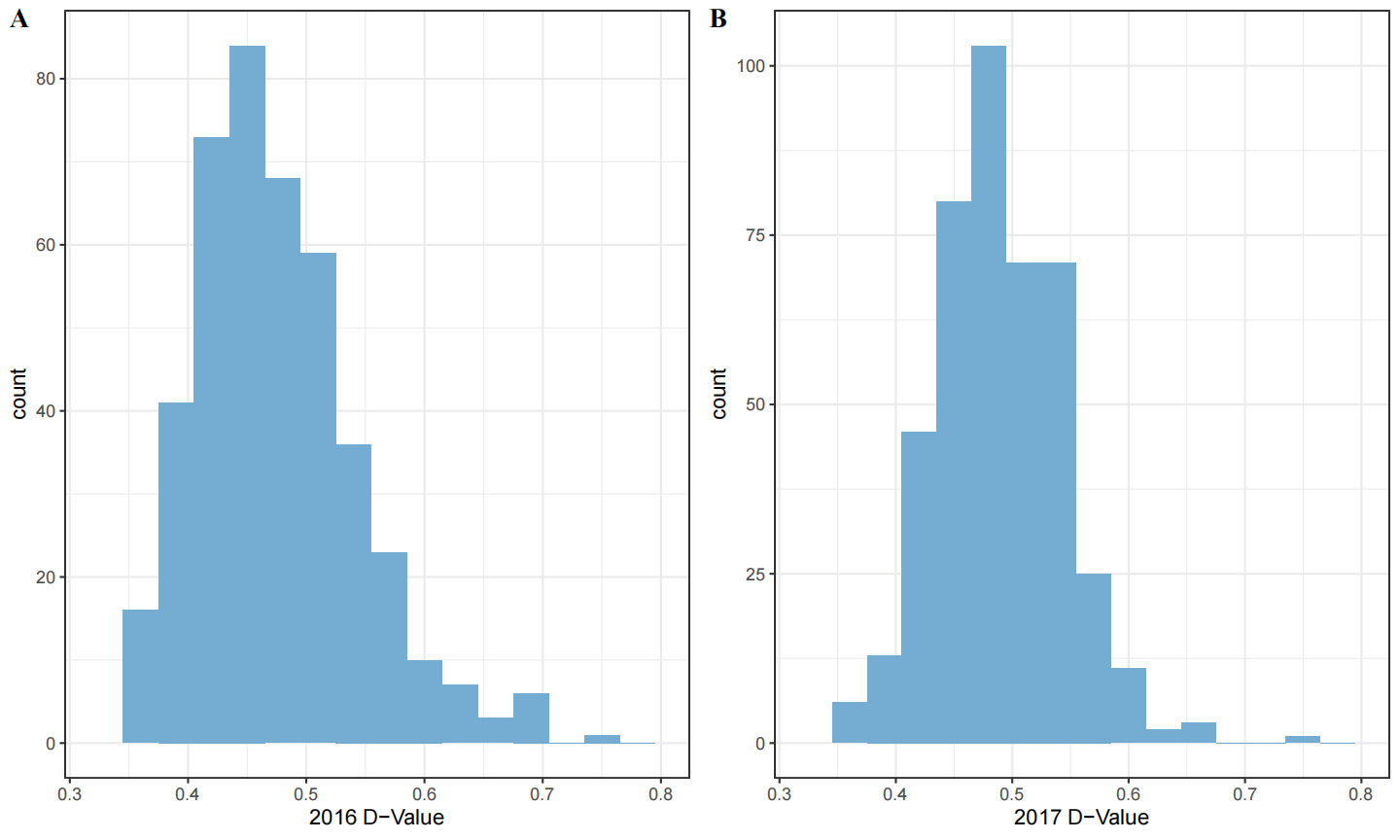


**Figure S3.** Histogram of comprehensive value: (A) 2016; (B) 2017.
